# Unlocking the Potential of Silver Nanoparticles: From Synthesis to Versatile Bio-Applications

**DOI:** 10.3390/pharmaceutics16091232

**Published:** 2024-09-21

**Authors:** Ahmad Almatroudi

**Affiliations:** Department of Medical Laboratories, College of Applied Medical Sciences, Qassim University, Buraydah 51452, Saudi Arabia; aamtrody@qu.edu.sa

**Keywords:** silver nanoparticles, synthesis methods, antimicrobial therapy, cancer therapy, other applications, bacteria

## Abstract

Silver nanoparticles (AgNPs) are leading the way in nanotechnological innovation, combining the captivating properties of silver with the accuracy of nanoscale engineering, thus revolutionizing material science. Three main techniques arise within the alchemical domains of AgNP genesis: chemical, physical, and biological synthesis. Each possesses its distinct form of magic for controlling size, shape, and scalability—key factors necessary for achieving expertise in the practical application of nanoparticles. The story unravels, describing the careful coordination of chemical reduction, the environmentally sensitive charm of green synthesis utilizing plant extracts, and the precise accuracy of physical techniques. AgNPs are highly praised in the field of healthcare for their powerful antibacterial characteristics. These little warriors display a wide-ranging attack against bacteria, fungi, parasites, and viruses. Their critical significance in combating hospital-acquired and surgical site infections is highly praised, serving as a beacon of hope in the fight against the challenging problem of antibiotic resistance. In addition to their ability to kill bacteria, AgNPs are also known to promote tissue regeneration and facilitate wound healing. The field of cancer has also observed the adaptability of AgNPs. The review documents their role as innovative carriers of drugs, specifically designed to target cancer cells with accuracy, minimizing harm to healthy tissues. Additionally, it explores their potential as cancer therapy or anticancer agents capable of disrupting the growth of tumors. In the food business, AgNPs are utilized to enhance the durability of packing materials and coatings by infusing them with their bactericidal properties. This results in improved food safety measures and a significant increase in the duration that products can be stored, thereby tackling the crucial issue of food preservation. This academic analysis recognizes the many difficulties that come with the creation and incorporation of AgNPs. This statement pertains to the evaluation of environmental factors and the effort to enhance synthetic processes. The review predicts future academic pursuits, envisioning progress that will enhance the usefulness of AgNPs and increase their importance from being new to becoming essential within the realms of science and industry. Besides, AgNPs are not only a subject of scholarly interest but also a crucial component in the continuous effort to tackle some of the most urgent health and conservation concerns of contemporary society. This review aims to explore the complex process of AgNP synthesis and highlight their numerous uses, with a special focus on their growing importance in the healthcare and food business sectors. This review invites the scientific community to explore the extensive possibilities of AgNPs in order to fully understand and utilize their potential.

## 1. Introduction

AgNPs have piqued significant interest and have undergone thorough investigation thanks to their unique physical and chemical characteristics [1]. Nanoparticles have a rich application history in various sectors, including medicine, food, healthcare, consumer items, and industry. In the medical field, AgNPs have been extensively studied for their therapeutic uses, demonstrating significant potential as angiogenesis inhibitors, anti-inflammatory medicines, agents that prevent tumor growth, and agents that combat bacterial infections [2]. The use of AgNPs in wound healing is attributed to their distinct physicochemical properties, which enhance their effectiveness in stimulating tissue regeneration and inhibiting infection [3]. Additionally, they have been scrutinized for their potential antiviral properties against various viruses, such as human immunodeficiency virus, hepatitis B virus, and respiratory syncytial virus [4].

Extensive research has been conducted on the medicinal applications and antibacterial properties of AgNPs. These nanoparticles can effectively combat bacteria, particularly antibiotic-resistant strains, in biomedical science [5]. Incorporating AgNPs into various materials, such as hydrocolloid impressions and mouthwashes, has significantly enhanced their antibacterial properties [6,7]. Additionally, they are widely utilized in wound dressings and topical treatments due to their remarkable antibacterial traits [8]. AgNPs have also demonstrated exceptional potential in cancer therapy, acting as antineoplastic drugs and inhibiting the proliferation of cancer cells [9]. Combined with traditional cancer medications, their efficacy increases [10]. Studies have also been conducted on their ability to regulate the growth and spread of breast cancer cells [11]. Much research has been dedicated to synthesizing and characterizing AgNPs, with green synthesis techniques utilizing plant extracts being a popular approach [12,13,14].

The characteristics of AgNPs, including their size, shape, and electric charge, have been extensively studied through advanced techniques such as UV-Vis spectroscopy and scanning electron microscopy [13]. These nanoparticles have demonstrated outstanding potential in a variety of applications. However, prior research has also thoroughly investigated their safety and potential toxicity to understand better how they disperse, are eliminated, and may impact animal subjects [15,16]. The synthesis of AgNPs in vivo from silver salts emphasizes the importance of comprehending their potential effects on biological systems [15]. Therefore, this review aims to provide a comprehensive overview of the historical development, synthesis techniques, and characterization methods of AgNPs, as well as their diverse applications in medicine, antibacterial properties, and cancer therapy. Additionally, the review delves into the biosafety and toxicity of AgNPs, including their distribution, elimination pathways, and potential effects on biological systems, to ensure their safe and effective use in various fields.

## 2. Synthesis of AgNPs

**Figure 1:**
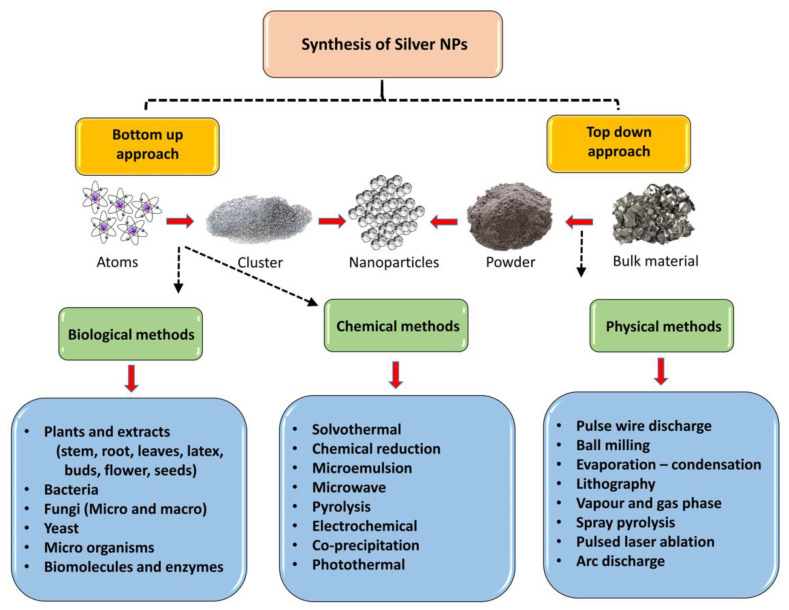
Synthesis of silver NPs by different biological, chemical, and physical methods.

Several methodologies are in practice for the synthesis of AgNPs, including biological methods, chemical methods, and physical methods (Figure 1). These synthesis methods are described as follows.

### 2.1. Biological Synthesis

**Figure 2:**
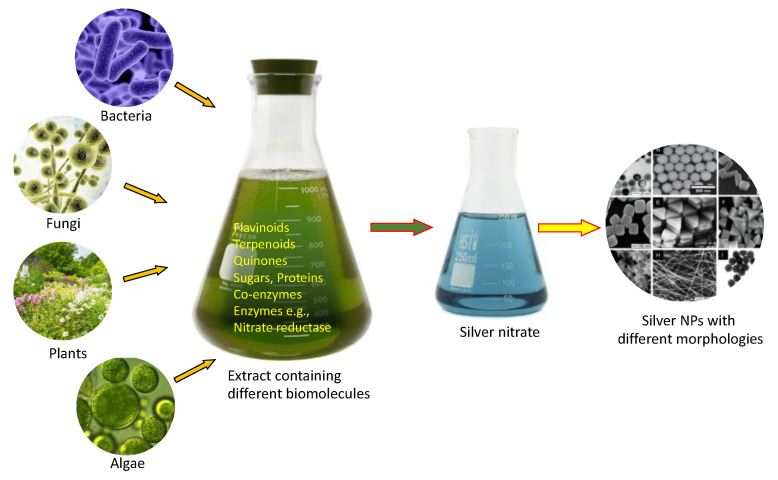
Synthesis of silver nanoparticles from different biological sources.

The green synthesis technique has surged in popularity for the biological production of AgNPs using microbes, plants, or plant extracts (Figure 2). Its eco-friendliness, cost-effectiveness, and ability to create unique nanoparticle properties such as size, shape, and surface characteristics are just a few of its benefits. Researchers have explored this technique and demonstrated its effectiveness in various fields, such as medicine, materials science, and environmental remediation. With precise control over nanoparticle production, exceptional optical, electrical, and catalytic properties can be exhibited, as evidenced by several studies [17,18,19].

The biosynthesis of AgNPs by microorganisms is a remarkable paradox: these particles, produced by specific bacteria, fungi, or algae, possess substantial antibacterial characteristics that are harmful to a broad spectrum of pathogens, while they do not cause any harm to the microorganisms responsible for their production. An appealing hypothesis is that the silver nanoparticles produced by microbes display targeted toxicity, specially designed against harmful cells [20]. This specificity may result from inherent disparities in the cellular architecture, composition of membranes, or metabolic pathways between the gene-producing microbes and the pathogens being targeted [21,22].

Furthermore, bacteria that produce AgNPs may have inherent resistance mechanisms that shield them from the potentially detrimental impacts of these nanoparticles. Potential resistance mechanisms involve synthesizing protective proteins, efflux pumps, or improved antioxidant defenses that counteract or alleviate the harmful impacts of AgNPs [23,24,25].

Moreover, the generating microorganisms may alter the surface chemistry of the biologically produced AgNPs to decrease their toxicity towards themselves and improve their selectivity against other organisms of interest. This process may include the covalent bonding of biomolecules, such as proteins, lipids, or polysaccharides, to the nanoparticles’ surfaces, potentially counteracting their harmful properties or modifying their interaction with various cell types [26,27].

- Synthesis of AgNPs based on Bacteria

Extensive research has been conducted on the potential of bacteria to produce AgNPs. Studies have shown that these microorganisms possess enzymes or proteins that effectively reduce silver ions and facilitate the creation of nanoparticles [19]. The biogenic synthesis technique, which utilizes bacterial cells or metabolites to convert silver ions into nanoparticles, has proven to be a highly efficient method for synthesizing AgNPs [28]. Moreover, the AgNPs produced by bacteria have potent antibacterial properties, making them highly suitable for diverse applications [29].

The bacteria-mediated synthesis of AgNPs is an eco-friendly approach that results in minimal waste production and high energy efficiency, rendering it environmentally sustainable [30]. The scientific community has extensively explored the bacterial-mediated synthesis of AgNPs, with bacterial proteins playing a crucial role in the production process [31].

The size and strength of the nanoparticles produced can vary depending on the bacterial strain used [29]. Different bacterial species, such as *Escherichia coli*, *Bacillus subtilis*, and *Streptococcus thermophilus*, have been utilized in the synthesis process, with parameters such as pH, temperature, and silver ion concentration being manipulated to optimize the process [32]. AgNPs synthesized by bacteria can be used beyond their antibacterial properties, with their potential applications in mosquito management also being explored [31]. Producing elemental silver through bio-fabrication using bacteria is a promising technique, with bacterial strains being easily distinguishable based on their antibacterial activity [33,34]. The potential applications of bacteria in producing AgNPs include agriculture, biomedicine, and environmental remediation [35,36,37]. This research opens up new opportunities for exploring the beneficial role of bacteria in various fields.

ii. Synthesis of AgNPs based on Fungi

The use of fungi in synthesizing AgNPs has gained significant attention due to their unique ability to produce enzymes that can reduce silver ions and stabilize resulting nanoparticles [38]. This area of research within nanotechnology is fascinating, as the biologically mediated production of nanoparticles through fungi is a simple, cost-effective, reliable, and eco-friendly approach [39].

Recent investigations have explored the potential of entomopathogenic fungi in producing AgNPs, with findings suggesting that these nanoparticles can be utilized as antibacterial agents and for managing insect pests [40]. Previous studies have also demonstrated the effectiveness of fungi such as *Aspergillus fumigatus*, *Aspergillus flavus*, and *Fusarium oxysporum* in producing AgNPs [41,42,43]. These investigations have highlighted the ability of fungi to reduce the concentration of silver ions and produce stable AgNPs of various sizes and shapes.

The use of fungi in the biogenic synthesis of AgNPs offers numerous advantages. This approach involves utilizing biomolecules sourced from organisms to act as reducing agents, coating agents, and stabilizers for AgNPs, known as green synthesis [44]. Compared to traditional chemical approaches, this method is cost-effective and environmentally friendly [1].

The synthesis of AgNPs through fungi offers a promising avenue for research. This involves using potent reducing agents generated by the fungi to convert silver ions into neutral atoms [14]. To enhance the efficiency of the process, it would be beneficial to optimize certain variables such as the fungal extract concentration, reaction duration, and temperature. This would lead to a streamlined process and ultimately improved outcomes [45].

**iii.**  **Synthesis of AgNPs based on yeast**

Through a biological method, AgNPs can be synthesized with the help of yeast. Studies have demonstrated the effectiveness of various yeast strains in producing AgNPs [46]. Synthesis involves converting silver ions into AgNPs using yeast cells or their extracts [47]. Yeast strains such as Saccharomyces cerevisiae, *Candida* sp., and *Meyerozyma guilliermondii* have all been utilized in creating AgNPs [47,48,49]. Moreover, researchers have explored combining yeast extracts with other substances, such as plant extracts or polymers, to produce AgNPs [50]. The uses of yeast-synthesized AgNPs go beyond their antibacterial properties and extend to their potential antioxidant properties and application in biomedicine [49]. Furthermore, researchers have also investigated the ability of yeast-mediated AgNPs to catalyze chemical reactions when exposed to light and their potential use in addressing environmental issues [47].

iv. Synthesis of AgNPs based on DNA

Recent research has revealed that DNA can be crucial in synthesizing AgNPs. By serving as a reducing agent, DNA’s intense attraction to silver ions enhances the stability of DNA, making it an effective template stabilizer.

The structural integrity of DNA after the reduction of silver ions is mainly determined by the interaction between DNA molecules and the resultant AgNPs. When silver ions (Ag^+^) are reduced in the presence of DNA, AgNPs are generated, capable of binding to DNA via various interaction mechanisms. These activities encompass the coordination with nitrogenous bases such as adenine, thymine, cytosine, and guanine, the binding to the phosphate backbone, and the intercalation through base pairs [51,52]. Interactions of this nature typically improve DNA stability by creating a protective structure around the DNA strands, preventing deterioration caused by environmental elements such as nucleases, heat, or chemical agents. The protective aspect of this phenomenon stems from the high affinity of silver ions and nanoparticles for the DNA structure, safeguarding the DNA against enzymatic breakage or denaturation [53,54,55]. Moreover, forming a DNA–AgNP complex can enhance the stability of the DNA molecule by preserving its structural integrity and inhibiting conformational alterations [56].

AgNPs have been successfully synthesized and observed to be located at the N-7 phosphate and guanine base pair on a DNA strand. These exciting developments have significant implications for advancing nanotechnology and its potential applications in various fields [57,58].

In addition, other studies have explored using calf thymus DNA as a template or reducing and stabilizing agent in producing AgNPs [55]. These investigations have focused on refining methods for synthesizing AgNPs and identifying their properties based on DNA. Such research could pave the way for discoveries and advancements in nanotechnology.

v. Green synthesis using plant extracts

The use of plant extracts for producing AgNPs, known as green synthesis, has become widely popular due to its sustainable and eco-friendly nature. Plant extracts contain various bioactive components such as flavonoids, phenols, terpenoids, and proteins that can act as reducing and stabilizing agents during the synthesis of AgNPs [59,60].

Various plant extracts such as *Tithonia diversifolia* [61], *Hygrophila auriculata* [62], *Acalypha wilkesiana* [63], *Diospyros lotus* [64], *Polygonatum graminifolium* [65], *Tridax procumbens* [66], *cauliflower* [67], *Euphorbia umbellate* [68], *Ficus cordata* [69], and others have been utilized to achieve the green synthesis of silver nanoparticles. These studies have showcased the potential of various plant extracts in synthesizing silver nanoparticles, demonstrating the versatility of this approach.

Not only is green synthesis cost-effective and environmentally safe, but it also offers unique benefits over traditional chemical procedures. The phytochemicals present in plant extracts can impart outstanding qualities to nanoparticles, such as improved stability, compatibility with living organisms, and antimicrobial properties [70,71,72]. Moreover, this method enables the production of nanoparticles with precise control over their size, shape, and surface characteristics by adjusting the composition of the plant extract and the reaction conditions [73,74]. However, it is essential to keep in mind that various factors such as the plant extract composition, reaction conditions, and the presence of other biomolecules can influence the green synthesis process [75]. Hence, optimizing these parameters is crucial to producing nanoparticles with desired properties and achieving consistent results.

vi. Bio-inspired synthesis

The field of bio-inspired synthesis is a captivating approach that takes cues from biological processes to design innovative materials and structures. Biomimicry involves replicating natural processes and structures to create novel materials with unique properties and capabilities. This technique has garnered significant attention in several fields, including energy storage, materials research, medicine, and catalysis. For instance, scientists have reported the development of hierarchical carbon materials for supercapacitors exhibiting exceptional performance using bio-inspired synthesis [76]. The study showcases an eco-friendly method of producing bio-inspired hierarchical carbon materials with porous architectures at the nanoscale and microscale, which function remarkably well as supercapacitor electrodes [76]. Bio-inspired synthesis has also been utilized to synthesize nanoparticles, such as AgNPs, which have been studied for various purposes, including antibacterial agents, catalysis, and medication administration [77]. Furthermore, bio-inspired synthesis has been extended to create materials with unique properties alongside nanoparticles. For example, researchers have developed hollow mesoporous bioactive glass nanoparticles using calcium carbonate as a solid template [78].

Bio-inspired synthesis has proven to be a highly effective method for producing hybrid materials with various biomedical applications. Researchers have been exploring the creation of biomimetic nanoparticles and materials, which offer significant promise for targeted drug delivery and imaging in various medical conditions. For example, a recent study by Dey et al. (2023) successfully synthesized doxorubicin-bioactive glass–ceramic hybrid nanoparticles using a bio-inspired approach. This breakthrough has paved the way for developing a highly effective anti-cancer therapy targeting specific cells and tissues [79]. Furthermore, biomimetic nanoparticles have been produced using leukocytes to enhance their targeting ability in cases of inflammation [80].

### 2.2. Chemical Synthesis

- Trisodium citrate AgNPs

Trisodium citrate is a highly effective substance in the production of AgNPs due to its ability to reduce and stabilize, which helps regulate the size and shape of the nanoparticles [81,82,83]. The use of trisodium citrate in the production of AgNPs has been extensively studied in various fields, including medicine, biology, chemistry, and materials science [81,83,84,85,86]. Researchers have explored using trisodium citrate in combination with other substances, such as tannic acid and borohydride, to achieve desired characteristics or improve the synthesis process [83,84] Additionally, trisodium citrate has been successfully used as a reducing agent in producing AgNPs in cotton fabric, enhancing its antibacterial and UV-protective properties [85].

In this method, silver nitrate (AgNO_3_) is commonly employed as this approach’s principal source of silver ions. Trisodium citrate has a double function: it acts as a reducing agent by reducing silver ions (Ag^+^) to metallic silver (Ag^0^), and it further acts as a capping agent by adsorbing onto the surface of the resultant nanoparticles to stabilize them [87,88]. This adsorption phenomenon induces electrostatic repulsion between the negatively charged layers of citrate, therefore inhibiting aggregation and improving the stability of colloidal particles. The stability of citrate-capped AgNPs is often maintained in aqueous solutions [89].

The stability of citrate-capped AgNPs in biological settings is affected by the development of a protein corona, a coating of biomolecules that adheres to the nanoparticle surface when it comes into touch with biological fluids [90]. The presence of a protein corona can modify the physicochemical characteristics of the nanoparticles, therefore impacting their stability, cellular incorporation, and possible toxicity. Including proteins in nanoparticles can stabilize them by steric hindrance and cause aggregation or increased dissolution, depending on the particular biomolecule interactions [91].

ii. Alanine/NaOH AgNPs

Alanine/NaOH AgNPs hold great promise in the field of nanotechnology. The nanoparticles are created by utilizing alanine and sodium hydroxide as crucial components. Through a highly efficient method, the conversion of silver ions (Ag^+^) into AgNPs is achieved with the help of alanine and NaOH [92]. The alkaline pH maintained by NaOH during synthesis aids the conversion of Ag^+^ ions into Ag NPs, making the process more efficient [93]. NaOH acts as a catalyst, expediting the reduction reaction and enhancing the synthesis process’s efficiency, which regulates the resulting AgNP dimensions, configuration, and strength [92,93].

The dimensions and morphology of the AgNPs can be adjusted based on several factors, such as alanine and NaOH levels, silver precursor, and reaction parameters, such as temperature and duration [92]. Alanine and NaOH have been studied extensively in producing AgNPs for various purposes, such as sensing, antibacterial properties, radiation detection, and catalytic activity [92,94].

In this method, the silver salt often employed in this technique is silver nitrate (AgNO_3_). When NaOH is present, alanine functions as both a reducing and a stabilizing agent. The elevated pH level induced by NaOH enables alanine’s deprotonation, increasing its ability to reduce carbon and facilitating the production of AgNPs [90,95]. Alanine molecules attached to the surface of the nanoparticles form steric hindrance, which inhibits aggregation [96]. Alanine-capped AgNPs exhibit very high biostability in biological settings because they include amino acids, naturally occurring biomolecules generally well tolerated by biological systems [97].

iii. Oleic acid AgNPs

Oleic acid AgNPs are a specific type of AgNPs that rely on oleic acid as a critical component in their production. The process involves using oleic acid as a reducing agent to convert silver ions (Ag^+^) into AgNPs [98,99,100]. Using oleic acid as a capping agent is crucial, as it prevents the nanoparticles from aggregating and enlarging, ultimately improving their stability and maintaining their colloidal stability [98,99].

In this approach, silver salts, namely silver acetate (AgC_2_H_3_O_2_), are frequently used. Oleic acid mainly stabilizes by creating a hydrophobic coating over the nanoparticles, inhibiting aggregation and improving their stability in organic solvents. The significant steric stabilization formed by the hydrophobic interactions among the oleic acid molecules on the nanoparticle surfaces renders these nanoparticles exceptionally stable in nonpolar liquids [101,102,103].

Within biological settings, the stability of oleic acid-capped AgNPs may be hindered by the hydrophobic characteristics of the oleic acid covering. In water-based biological solutions, the layer of oleic acid can desorb or have unfavorable interactions with biomolecules, which may result in aggregation or fast dissolution [104].

iv. Carbonyl-containing reducing sugars

Carbonyl-containing reducing sugars, including aldehydes or ketones, are increasingly used as economically friendly reducing agents. These sugars can undergo electron donation to silver ions (Ag⁺), therefore enabling their reduction to metallic silver (Ag^0^) and the resulting production of nanoparticles [105]. Primarily used reducing sugars in this procedure are glucose, fructose, maltose, sucrose (after hydrolysis), lactose, and ribose. Glucose, a monosaccharide containing an aldehyde group, is extensively utilized because of its plentiful availability, inexpensive nature, and efficient capacity for reduction [106,107].

Utilizing reducing sugars in nanoparticle manufacturing has numerous benefits. Non-toxic, biodegradable, and presenting little environmental hazards compared to traditional chemical reducing agents such as sodium borohydride or hydrazine, these sugars are an appealing choice for green synthesis [96,108]. Moreover, nanoparticles produced by synthesizing reducing sugars demonstrate enhanced biocompatibility, rendering them appropriate for various biological uses. In addition to their absorption properties, these sugars also serve as capping agents, stabilizing the nanoparticles and inhibiting aggregation, essential for preserving their colloidal stability [96,109].

While several established chemical techniques exist for creating AgNPs using various reducing agents, researchers have found that this method produces highly stable nanoparticles, as depicted in Table 1:

1. Chemical reduction: The synthesis of AgNPs involves the reduction of silver ions using chemical reducing agents such as sodium borohydride, sodium citrate, ascorbic acid, or polyol techniques [1,110];
2. Electrochemical procedures involve reducing silver ions by using an electrode as the reducing agent [110];
3. Photochemical reduction: AgNPs can be produced by exposing a solution containing silver ions to ultraviolet (UV) light or other forms of radiation [111];
4. Microwave-assisted synthesis: Microwave irradiation can expedite the reduction of silver ions and facilitate the production of AgNPs [112];
5. The sol–gel approach involves integrating silver precursors into a sol–gel matrix and then reducing them to generate AgNPs [1].

The chemical approaches precisely manipulate the dimensions, morphology, and durability of the AgNPs that are produced. The selection of the appropriate technique relies on various aspects, including the desired characteristics of the nanoparticles, the capacity to scale up production, and the simplicity of the synthesis process.

### 2.3. Physical Methods

- Pulse Wire Discharge

Pulse wire discharge is a synthesis technique in which a high-voltage pulse is directly applied to a tiny wire, typically composed of silver. Rapid heating of the wire by the electrical solid discharge leads to vaporization and condensation into nanoparticles when cooled [113,114]. The discharge parameters, including voltage, current, and the surrounding media, determine the potential of this approach to generate nanoparticles of various sizes and forms [115]. One advantage of pulse wire discharge is its ability to generate substantial amounts of nanoparticles without chemical additions rapidly. Nevertheless, that approach necessitates meticulous electrical parameters and specialized apparatus regulation to manage high voltages [116].

ii. Ball milling

Ball milling is a mechanical procedure that entails the preparation of bulk silver into nanoparticles by immersing silver material in a cylindrical chamber filled with rigid balls, usually composed of ceramic or steel. The spinning induces recurrent collisions between the balls and the silver substance, causing disintegration into smaller particles [117,118]. Modulating the milling duration, speed, and ball size allows for precise control over the dimensions and morphology of the nanoparticles [118]. Ball milling is an economical and uncomplicated technique well-suited for mass industrial production. Introducing contaminants from the milling media necessitates meticulous monitoring to prevent oxidation and contamination [119,120].

iii. Evaporation–condensation

Evaporation–condensation is a technique by which silver is heated in a vacuum or inert gas atmosphere to cause the metal to evaporate. Subsequently, the metal condenses into nanoparticles following cooling [121]. This procedure necessitates using a carrier gas to convey the vaporized silver and a regulated temperature gradient to promote condensation. This approach enables the production of nanoparticles with a limited range of sizes and excellent particle size regulation. Nevertheless, the process necessitates elevated temperatures and specialized vacuum infrastructure, reducing its viability for economically efficient large-scale manufacturing [122,123].

iv. Lithographic printing

Lithography is a widely employed method in microelectronics, directly applicable to producing silver nanoparticles. This technique involves applying a photoresist layer onto a substrate, which is subsequently subjected to a specific pattern of light to form a mask. The exposed regions undergo development, upon which silver is deposited into the substrate. Once the mask is removed, it leaves behind meticulously designed silver nanoparticles [124,125]. Lithography provides exceptional nanoparticle shape and size manipulation, rendering it well-suited for applications that demand accurate patterning at the nanoscale [126]. Nevertheless, it is an intricate and laborious procedure that necessitates costly technologies and could be more optimal for large-scale nanoparticle synthesis [127].

v. Vapor and Gas Phase Methods

Vapor and gas phase techniques refer to producing silver nanoparticles from silver vapor under a regulated gas atmosphere. Standard techniques in this category include chemical vapor deposition (CVD) and physical vapor deposition (PVD). These techniques include the transportation of silver vapor in a carrier gas, whereby nanoparticles are generated while the vapor undergoes cooling and condensation [128,129]. These methods offer precise manipulation of particle size and shape, producing nanoparticles with exceptional purity [130]. Nevertheless, their scalability and cost-effectiveness for large-scale production could be much better due to the need for high temperatures, vacuum conditions, and advanced equipment [131].

vi. Spray pyrolysis

Spray pyrolysis involves atomizing a solution carrying silver precursors into tiny droplets employing a spray nozzle. Subsequently, these droplets are processed in a high-temperature furnace, where they experience swift evaporation and breakdown, producing silver nanoparticles [132]. Spray pyrolysis’s advantage is its ability to enable the continuous and scalable synthesis of nanoparticles with precise control over their size and content. This approach is adaptable and can be employed to generate a diverse array of nanoparticle compositions. Controlling the size distribution and preventing agglomeration can be formidable tasks [132,133].

vii. Pulsed laser ablation

Pulsed laser ablation is a method by which a high-energy laser beam is concentrated onto a silver target immersed in a liquid media. High-intensity laser pulses induce vaporization of the material, forming a plasma plume that subsequently cools and condenses into nanoparticles. The manipulation of laser parameters, including wavelength, pulse duration, and energy, allows for the regulation of the size and shape of the nanoparticles [134,135]. A key feature of pulsed laser ablation is its ability to generate nanoparticles of high purity without the requirement of chemical additions [135]. Nevertheless, this procedure can be very demanding regarding energy consumption.

viii. Arc discharge

Arc discharge is the process of generating a high-energy electric arc between two silver electrodes immersed in a liquid media in a gas environment. The arc’s heat induces vaporization of the silver, which then condenses to produce nanoparticles [136]. The arc conditions, including voltage, current, and surrounding media, determine the diverse nanoparticle sizes and shapes this approach can generate. Arc discharge is a straightforward and reliable method suitable for large-scale manufacturing. Nevertheless, regulating the distribution of particle sizes might be difficult, and the procedure may introduce contaminants from the electrode materials [137,138].

### 2.4. A Comparative Analysis of Green Synthesis and Conventional Chemical Methods

Green synthesis is often more economically efficient as it utilizes low-cost, regenerable raw resources such as plant extracts and microorganisms. Many of these activities occur at average ambient temperatures and pressures, which helps to decrease energy usage and operational expenses [139,140]. By comparison, conventional chemical techniques may necessitate expensive chemicals and substantial energy inputs to sustain precise temperatures and pressures [141,142].

Conventional chemical techniques have always been preferred for their capacity to generate nanoparticles of high uniformity, with meticulous regulation of size and shape [81,82,83]. Nevertheless, recent progress in green synthesis has attained comparable precision, frequently generating nanoparticles within the targeted size range of 1–100 nanometers [143]. One of the distinctive benefits of green synthesis is the ability to integrate biological molecules onto the surfaces of nanoparticles, improving their performance for particular uses such as medication delivery or biosensing. Nevertheless, the diversity in biological extracts employed in green synthesis might occasionally result in variations in the surface properties of nanoparticles [144,145]. Conventional approaches rely on synthetic capping agents, contributing to more reliable outcomes. Although conventional techniques are highly suitable for large-scale production because of their proven capacity to be scaled up, continuous research enhances the scalability and industrial feasibility of green synthesis, establishing it as a competitive option (Table 1).

**Table 1:** Techniques for producing uniform and near-spherical AgNPs.

Method	Description	Example	Capping Agent	Experimental Conditions	Key Characteristics	Reference
Chemical Reduction	Reduction of silver salt using a reducing agent and stabilizer	Turkevich: Silver nitrate + sodium citrate	Sodium citrate	Aqueous, heated to boiling	Precise control, uniform size, toxic byproducts	[141,142]
Polyol Method	Polyol as a solvent and reducing agent	Silver nitrate in ethylene glycol	Polyvinylpyrrolidone (PVP)	Ethylene glycol, heated	Size control, not always environmentally benign	[146,147]
Microwave-Assisted Synthesis	Microwave irradiation for rapid synthesis	Silver nitrate and glucose solution	None (Glucose acts as both)	Microwave irradiation	Rapid, moderate control, minimal harmful chemicals, equipment needed	[148,149]
Green Synthesis	Eco-friendly reducing agents	Neem extract reducing silver nitrate	Phytochemicals from plant extract	Room temperature, aqueous	Environmentally friendly, cost-effective, scalability challenges	[139,140]
Sol–gel Method	Transformation into a gel incorporating nanoparticles	Silver nitrate with sol–gel precursor forms AgNP gel	None specified	Sol–gel process	Controlled synthesis in a gel matrix, energy-intensive	[150,151]

### 2.5. The Factors That Influence the Synthesis of AgNPs

**Figure 3:**
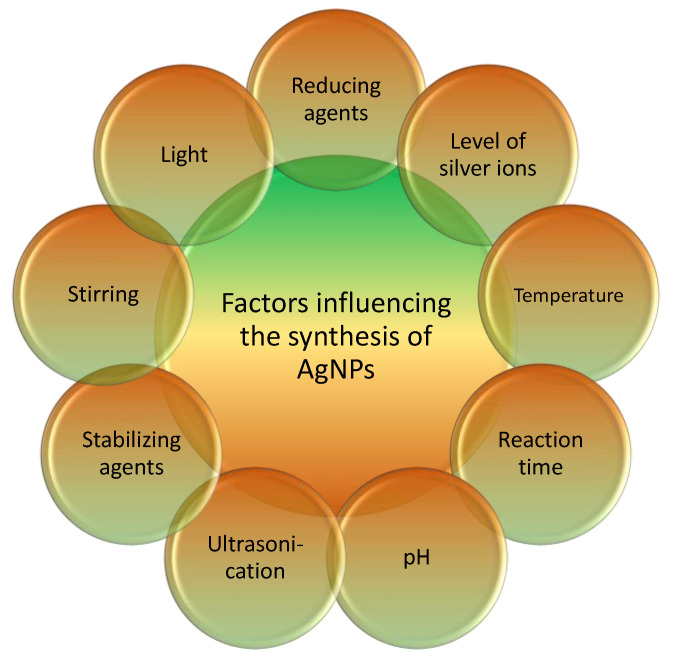
Different physical and chemical factors influencing the synthesis of silver nanoparticles.

Synthesizing AgNPs is a complex undertaking influenced by various factors depending on the chosen method and conditions. Within the literature, several elements have been identified that can impact the synthesis process and alter the resulting nanoparticle characteristics (Figure 3). In order to improve the process and achieve high-quality nanoparticles, researchers often study and analyze the fundamental factors that contribute to the creation of AgNPs. These factors typically include the following:

1. Reducing Agent: The choice of reducing agent or extract used in the synthesis process is crucial. Various plant extracts or biological agents can serve as reducing agents to transform silver ions into AgNPs [14,152]. The reducing agent’s content and concentration can affect the nanoparticles’ dimensions, morphology, and durability [152,153];
2. Concentration of Silver Ions: The amount of silver ions (Ag^+^) in the reaction mixture is another critical component. The presence of Ag^+^ ions can influence the nucleation and development of nanoparticles, resulting in their size and form changes. The ideal condition for synthesizing AgNPs is a concentration of 1 mM of Ag^+^ [18];
3. The temperature plays a significant role in the formation of AgNPs. It impacts the rate of the reduction reaction and the synthesis of the particles. According to Heydari and Rashidipour (2015), higher temperatures can speed up the reduction process and facilitate the formation and enlargement of nanoparticles. However, excessively elevated temperatures can lead to agglomerates or unwanted enlargement of particles. The ideal temperature for creating AgNPs may vary depending on the technique and circumstances [18];
4. The reaction time is an important parameter that influences the synthesis of AgNPs. The time the reaction takes dictates the degree of reduction and the range of sizes of the nanoparticles. Longer reaction durations tend to increase the size of nanoparticles, while shorter reaction times can result in smaller particles [154]. Additionally, the reaction time affects the stability and yield of the nanoparticles [154];
5. The pH level is a critical factor that can impact the production of AgNPs. Various pH values can affect the reduction rate, stability, and size distribution of the nanoparticles. The ideal setting for producing AgNPs is an alkaline pH of 9 [18];
6. Light: Exposure to light can significantly impact the creation and unique properties of AgNPs. Extensive research has explored how light serves as an energy source and influences both the reduction reaction and formation of nanoparticles [155]. Additionally, studies have investigated the effects of light exposure on the production of AgNPs using various plant extracts, such as *Solanum xanthocarpum* L. berry extract and *Catharanthus roseus* Linn. *Leaves and Ocimum bacilicum* L. leaf extract [153]. Furthermore, researchers have utilized light exposure to assess the photocatalytic activity of AgNPs. These nanoparticles were tested for their ability to decompose methylene blue when exposed to sunlight, highlighting their impressive photocatalytic properties [156];
7. Stirring, ultrasonication, and stabilizing agents are crucial factors in producing and preserving AgNPs. Several sources provide valuable insights into these elements. For example, Suci et al. (2022) conducted a study that examined the influence of ultrasonication and stirring on the dispersion of AgNPs. The study found that both procedures contribute to creating a homogeneous distribution of nanoparticles [157]. Similarly, Ameh et al. (2022) outlined a process for creating AgNPs that are stabilized by cetyltrimethylammonium bromide. The study emphasized the use of intense magnetic stirring as a method to enhance the stability of nanoparticles [158].

## 3. Characterization of AgNPs

Numerous types of characterization of AgNPs are discusse (Table 2) as follows:

### 3.1. Physical Characterization Techniques

#### 3.1.1. UV-Visible Spectroscopy

UV-visible spectroscopy is a commonly used method for analyzing AgNPs. This involves measuring the absorption and scattering of light within the UV-visible spectrum. The technique comprises several vital components meticulously integrated to ensure precise and dependable results.

- Absorption Measurement: UV-visible spectroscopy is used to measure light absorption by AgNPs, with their size, shape, and concentration affecting the results. The absorption spectrum typically displays a visible peak known as the surface plasmon resonance (SPR) peak, corresponding to the collective movement of conduction electrons within the nanoparticles [168,169];
- When examining AgNPs, analyzing the location and intensity of the SPR peak in the UV-visible spectrum can provide valuable information about their size and concentration. Generally, larger nanoparticles will cause the peak to shift toward longer wavelengths, while smaller ones will shift toward shorter wavelengths. Additionally, the strength of the peak directly corresponds to the quantity of nanoparticles within the sample [168,169];
- Analysis of Shape: By examining the shape of the absorption spectra, it is possible to deduce the morphology and structure of the AgNPs. Various geometrical shapes, such as spheres, rods, or triangles, have distinct absorption characteristics. For instance, anisotropic nanoparticles such as silver nanorods have several absorption peaks due to their unique optical properties [168,169];
- Determining Quantity: UV-visible spectroscopy can calculate the concentration of AgNPs in a sample. This is carried out by comparing the absorbance at the surface plasmon resonance (SPR) peak with a calibration curve generated using standard solutions with known concentrations [170].

Sekar et al. (2012) utilized UV-Vis spectroscopy to examine the absorption spectra of AgNPs produced using Catharanthus roseus leaves. The absorption peak confirmed AgNPs and their reduction from silver ions at 448 nm [17]. Similarly, Moond et al. (2023) employed UV-Vis analysis to evaluate AgNPs produced by Trigonella foenum-graecum leaf extract biosynthesis. The detection of an absorption peak at 460 nm confirmed the existence of AgNPs and suggested surface plasmon resonance phenomena [171].

UV-visible spectroscopy is an effective way to observe AgNPs’ long-term stability and aggregation tendencies. Displacements or widening of the SPR peak in the absorption spectrum may indicate nanoparticle aggregation or changes in their size or shape [168,169].

#### 3.1.2. Transmission Electron Microscopy (TEM)

Transmission electron microscopy (TEM) is a highly efficient method for analyzing AgNPs, providing high-resolution imaging and precise structural data at the nanoscale. Here are some critical aspects of transmission electron microscopy (TEM) about AgNPs:

- Imaging: TEM allows for direct observation of AgNPs, offering valuable insights into their dimensions, structure, and form. The advanced imaging capabilities of TEM enable the direct visualization of individual nanoparticles and the analysis of their internal structure [172,173];
- TEM images can be utilized to measure the dimensions and morphology of AgNPs. The analysis of these images allows for determining the nanoparticles’ average size, size distribution, and aspect ratio [174,175];
- Crystallographic Analysis: TEM can provide valuable information about the crystallographic structure of AgNPs through crystallographic analysis. Using selected area electron diffraction (SAED) patterns obtained from TEM images makes it possible to identify the crystal lattice and determine the crystallinity of nanoparticles [154,176];
- Elemental Analysis: The elemental makeup of AgNPs can be examined using energy-dispersive X-ray spectroscopy (EDS) along with TEM. EDS facilitates the detection and mapping of silver atoms’ spatial arrangement within the nanoparticles [177,178];
- Surface Analysis: Utilizing Transmission Electron Microscopy (TEM) may provide valuable insights into the surface characteristics of AgNPs. Through high-resolution imaging of the nanoparticle surface, one can gather valuable information regarding surface roughness, flaws, and surface coatings [179,180].

TEM provides a comprehensive means of studying AgNPs’ clustering and clustering behavior. By visualizing the nanoparticles under different conditions or following specific procedures, one can gain insight into the degree of aggregation and the interactions between the nanoparticles [181,182].

#### 3.1.3. Scanning Electron Microscopy (SEM)

The synthesis and evaluation of AgNPs have been extensively explored through SEM in conjunction with various other techniques. SEM has enabled researchers to visualize the size and shape of the produced AgNPs, providing valuable insights into their structural properties [160,183,184]. Through SEM analysis, researchers have observed that AgNPs have a spherical shape and a size distribution ranging from 0 to 50 nm, 10 to 46 nm, and 25 to 50 nm. This highlights the effectiveness of this technique in accurately characterizing the physical characteristics of AgNPs [160,183,185,186].

#### 3.1.4. X-ray Diffraction (XRD)

When examining AgNPs, X-ray diffraction (XRD) is a highly utilized method for analyzing their characteristics. This technique yields valuable information about their crystal structure, phase composition, and degree of crystallization. Listed below are several fundamental components of XRD as it relates to AgNPs:

- Crystal Structure: XRD is a technique that enables the analysis of the diffraction pattern obtained from X-ray interaction with the crystal lattice. This technique is employed to identify the crystal structure of AgNPs. Information regarding the atomic configuration within the nanoparticles can be obtained by examining the spatial distribution and magnitudes of the diffraction peaks [177];
- Phase Identification: XRD can discern the distinct phases within the AgNPs. The phases of the nanoparticles can be detected by comparing the diffraction pattern with reference patterns from a database [187];
- Crystallite Size: The XRD technique can be utilized to determine the average dimensions of AgNPs by analyzing the broadening of the diffraction peaks. Employing the Scherrer equation or other relevant methods makes it possible to compute the size of crystallites by examining the extent of peak broadening, as previously demonstrated [188];
- Crystallinity: XRD can determine the level of crystallinity exhibited by the AgNPs. The strength and sharpness of the diffraction peaks offer insights into the organization and quality of the crystal lattice [189];
- Phase Transformation is a powerful tool for examining the structural properties of AgNPs, including their crystal structure, phase composition, degree of crystallinity, and any phase changes that may occur. By subjecting the nanoparticles to temperature, pressure, or chemical treatments, XRD can identify phase transformations or alterations in their crystal structure. The presence or absence of diffraction peaks is a vital indicator of phase transitions [190].

#### 3.1.5. Energy-Dispersive X-ray Spectroscopy (EDS)

In nanoparticle research, AgNP analysis provides compelling evidence of today’s advanced analytical methods. Energy-Dispersive X-ray Spectroscopy (EDS) is a crucial approach that offers an elemental profile of nanoparticles, ensuring the accuracy of their composition [191,192]. EDS’s ability to accurately detect and measure the elemental components of AgNPs is crucial in characterizing nanoparticles. When used in conjunction with other characterization techniques such as TEM, SEM, UV-visible spectroscopy, XRD, and FTIR-ATR, EDS yields a harmonious ensemble of analytical insights, revealing the physical, structural, and chemical characteristics of AgNPs and provides a comprehensive understanding of their nanoscale properties [193,194].

The exceptional specificity of EDS in identifying elemental silver is remarkable, confirming the successful production of AgNPs and their purity. EDS analysis goes beyond simple characterization and explores the domain of functional performance. Identifying metallic silver peaks by EDS is a strong indicator of reliability, establishing a reference point for the antibacterial effectiveness of AgNPs [192,195].

#### 3.1.6. Dynamic Light Scattering (DLS)

Dynamic Light Scattering (DLS) is a widely used technique for determining the size and distribution of nanoparticles in a liquid medium [196]. DLS provides data on their size and heterogeneity by measuring the amount of light scattered by particles in a liquid [196]. DLS has been utilized in numerous studies to characterize AgNPs generated through different procedures and extracts. Researchers have applied DLS to analyze the size, size distribution, and zeta potential of AgNPs produced using various plant extracts, such as *Piper betle* L., *Piper sarmentosum* Roxb., *Gymnema sylvestre*, *Salvia miltiorrhiza*, *Momordica charantia*, *Annona reticulata*, *Azadirachta indica*, *Pisonia alba*, and *Curcuma longa* [197,198,199,200,201]. The investigations demonstrated the effectiveness of utilizing DLS to characterize the synthesized AgNPs, providing valuable insights into their stability and dimensions. DLS has been employed to examine the interaction between AgNPs and various substances, including lectins, polymer glycoconjugates, and plant extracts [202].

#### 3.1.7. Atomic Force Microscopy (AFM)

Atomic Force Microscopy (AFM) is a highly effective method to investigate the surface morphology and mechanical characteristics of materials at the nanoscale [203]. This involves moving a pointed probe across the surface of a sample and quantifying the forces between the probe and the sample [203]. AFM has been extensively used to characterize AgNPs produced through diverse procedures and extracts. In a study by Paulkumar et al. (2020), AFM was employed to ascertain the dimensions and morphology of AgNPs produced using Aerva lanata flower extract. The AFM research revealed many characteristics of the produced AgNPs, yielding valuable insights into their morphology [204].

Additionally, AFM was used to analyze the dimensions and morphology of AgNPs produced from calli cells of *Citrullus colocynthis*. The AFM images revealed that the produced AgNPs had a mostly spherical morphology, with an average diameter of 31 nm [205].

The analysis of surface defects and crystal structure as well as size and shape characterization of AgNPs has been made possible using AFM. High-resolution imaging using AFM has allowed for the examination of crystal defects, such as stacking faults and point defects, on the surfaces of Ag nanoplates. This examination has revealed a significant presence of crystal defects, which provides valuable information about their surface reactivity [206].

#### 3.1.8. Zeta Potential Analysis

Zeta potential analysis is a valuable technique used to measure nanoparticles’ surface charge and stability, such as AgNPs [207,208,209]. By examining the electrostatic repulsion between particles, zeta potential provides essential information for understanding their stability and potential applications [210].

Numerous studies have utilized zeta potential analysis to characterize AgNPs produced through various procedures and extracts. For instance, zeta potential analysis was employed to determine the surface charge and stability of AgNPs generated using the flower extract of Aerva lanata [208]. This analysis yielded valuable insights into the stability of the produced AgNPs. Additionally, zeta potential analysis was used to evaluate the stability of AgNPs created from the fruit extract of Rhus criteria, indicating the stability of silver particles manufactured using green methods [208].

The surface charge of AgNPs and its impact on biological activities and stability assessment were examined using zeta potential analysis. The surface charge of AgNPs, synthesized with ginger spent, was determined through zeta potential analysis [211], providing valuable insight into their interactions with biological systems. Zeta potential analysis was also conducted on AgNPs produced with Pterocarpus marsupium to assess their surface potential and stability [167].

### 3.2. Chemical Characterization Techniques

Understanding the properties and composition of AgNPs is crucial, and chemical characterization techniques are essential for achieving this. These methods offer valuable insights into the chemical makeup, purity, and functional groups present in the nanoparticles. Spectroscopic techniques, chromatographic techniques, and mass spectrometry are some of the ways to analyze AgNPs chemically. These approaches provide valuable information on the nanoparticles’ dimensions, morphology, composition, and surface characteristics.

#### 3.2.1. High-Performance Liquid Chromatography

High-performance liquid chromatography (HPLC) is an effective technique for separating and analyzing the components in a silver nanoparticle sample. By coupling HPLC with detection methods such as UV-visible spectroscopy or mass spectrometry, a more comprehensive understanding of the nanoparticles can be gained, including information on their composition and surface properties [1].

When examining AgNPs, mass spectrometry is a practical option. This method can provide valuable information on the composition, size, and organization of the nanoparticles and their interactions with biological systems [212]. Multiple mass spectrometry techniques, such as inductively coupled plasma mass spectrometry (ICP-MS) and matrix-assisted laser desorption/ionization mass spectrometry (MALDI-MS), can be utilized for this analysis [15,213]. ICP-MS is commonly used for precise measurements of AgNPs, whereas MALDI-MS can offer insights into the organization of the nanoparticles in tissues [213].

#### 3.2.2. Fourier Transform Infrared Spectroscopy (FTIR)

FTIR spectroscopy is a precious analytical technique for identifying and characterizing various materials, including AgNPs. The method involves several essential components:

1. Molecular Identification: FTIR spectroscopy can determine the molecular composition of a sample by measuring the absorption and transmission of infrared light. This technique can detect and analyze the functional groups and chemical bonds present in the sample, enabling the identification of various substances [214,215];
2. Analysis of Chemical Structure: FTIR spectroscopy provides valuable information on the chemical composition of AgNPs. By comparing the FTIR spectrum of the nanoparticles to reference spectra, it is possible to identify specific functional groups or surface coatings on the nanoparticles [216,217];
3. Surface Chemistry Examination: FTIR spectroscopy allows for examining the chemical composition of the surface of AgNPs, providing valuable insights into surface functional groups, adsorbed species, and chemical changes [218,219];
4. Quantitative Analysis: FTIR spectroscopy can perform quantitative analysis on AgNPs, estimating the nanoparticle concentration in a given sample by establishing a relationship between the strength of specific absorption patterns and the number of nanoparticles present [220,221];
5. Investigation of Stability and Interactions: FTIR spectroscopy can investigate the stability and interactions of AgNPs. Analyzing variations in the FTIR spectrum over time or in different situations makes it possible to evaluate the stability of the nanoparticles and examine their interactions with other molecules or surfaces [222,223].

#### 3.2.3. Raman Spectroscopy

Silver nanoparticles can be effectively characterized by Raman spectroscopy, particularly when augmented by Surface-enhanced Raman Scattering (SERS). Surface-enhanced Raman scattering (SERS) occurs when molecules are adsorbed onto the surface of silver nanoparticles, which substantially amplifies the Raman signal [224]. This characteristic renders Raman spectroscopy highly valuable for investigating the surface chemistry and molecular interactions occurring at the surface of nanoparticles. With its exceptional sensitivity to variations in the immediate surroundings, this technology is well-suited for detecting minute quantities of adsorbed species. Consequently, it is an excellent instrument for investigating [225].

#### 3.2.4. X-ray Photoelectron Spectroscopy (XPS)

X-ray Photoelectron Spectroscopy (XPS) is a very effective analytical method that accurately determines silver nanoparticle’s elemental composition and chemical state on their surface. The XPS technique may identify oxidation states, impurities, and surface coatings, therefore offering valuable information on the surface chemistry and possible reactivity of the nanoparticles [226,227]. Precise knowledge is essential for applications in which surface characteristics directly impact the effectiveness of silver nanoparticles, such as in catalysis, antibacterial coatings, and sensors.

#### 3.2.5. Thermal Analysis Techniques

Advanced thermal analysis methods, including Thermogravimetric Analysis (TGA), Differential Scanning Calorimetry (DSC), and Differential Thermal Analysis (DTA), are essential for comprehending the thermal characteristics and dynamics of AgNPs [228]. TGA quantifies mass variations concerning temperature, yielding significant insights into the temperatures at which decomposition occurs, oxidation stability, and organic capping agents or other stabilizers on the nanoparticles’ surface [229,230]. The significance of this research is especially pronounced in applications that necessitate AgNPs to preserve their structural integrity and functional characteristics with changing heat conditions, such as in the fields of catalysis and electronics.

DSC enhanced TGA by quantifying the thermal transport related to nanoparticle transitions, including melting, crystallization, and phase shifts. Discontinuous scanning calorimetry offers valuable information on AgNPs’ thermal stability, phase purity, and heat capacity. These properties are crucial for comprehending their performance in high-temperature applications [231,232]. Optimization of thermal processing parameters for AgNPs-based materials and assurance of their performance in thermal management and conductive applications heavily rely on the information acquired from DSC.

DTA significantly identifies exothermic and endothermic processes, including oxidation, reduction, and phase changes, in AgNPs [233]. This experimental method offers supplementary data regarding the thermal stability and chemical reactivity of AgNPs, therefore proving valuable in the development of materials that necessitate meticulous regulation of temperature and stability [234].

## 4. Overview of Biomedical Applications

**Figure 4:**
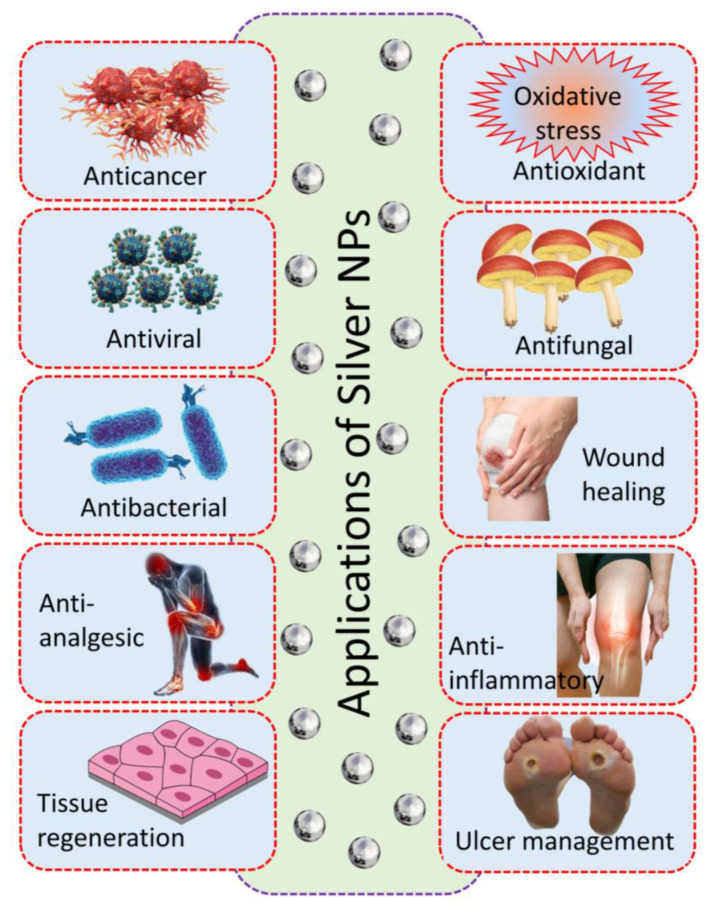
Applications of silver NPs in different health management fields.

AgNPs have been extensively researched and analyzed due to their unique properties and potential applications in various fields, including biomedicine [5]. AgNPs have gained significant attention in recent years for their promising features and versatile uses in the biomedical industry (Table 3 and Figure 4).

### 4.1. Wound Healing

AgNPs have shown great promise in wound healing due to their unique qualities, such as antibacterial activity, anti-inflammatory characteristics, and ability to promote tissue regeneration. Studies have demonstrated that the antibacterial properties of AgNPs aid in preventing infection and reducing microbial presence at the wound site, playing a critical role in successful wound healing [7,249,290]. Additionally, AgNPs have been found to regulate the inflammatory response, promoting a harmonized immune reaction and reducing excessive inflammation [290]. This creates an optimal environment for wound healing. Research has also shown that AgNPs can enhance the development of granulation tissue, a crucial component in wound healing [250]. They facilitate the movement and rapid growth of fibroblasts, endothelial cells, and keratinocytes, creating new tissue and restoring the extracellular matrix [250]. Studies have shown that AgNPs can accelerate the process of re-epithelialization, which involves the migration and rapid growth of epithelial cells to cover the wound area [251]. This helps speed up wound healing and promote the restoration of the skin’s protective barrier. Moreover, AgNPs have been incorporated into various wound dressings and scaffolds to sustain the release of silver ions, enhancing their antimicrobial and wound-healing properties [252]. These dressings prevent infections, promote tissue regeneration, and maintain a moist wound environment.

During the inflammation phase, AgNPs mitigate excessive inflammation by decreasing pro-inflammatory cytokines such as TNF-α, IL-6, and IL-1β. During the proliferation phase, AgNPs stimulate fibroblast activity and increase collagen production, which is essential for tissue regeneration and wound healing. The remodeling phase involves the regulation of matrix metalloproteinases (MMPs), which are responsible for remodeling the extracellular matrix (ECM) [291,292].

Moreover, AgNPs enhance angiogenesis by increasing the expression of vascular endothelial growth factor (VEGF), facilitating the proliferation of new blood vessels, and enhancing the transportation of oxygen and nutrients to the site of injury. Moreover, they regulate cell signaling pathways, including PI3K/Akt and MAPK/ERK, thereby augmenting cell survival, proliferation, and migration essential for tissue regeneration [293,294].

However, it is essential to note that while AgNPs have shown promising results in wound healing, their safety, and potential cytotoxicity need to be thoroughly evaluated. Previous research has examined their compatibility with living organisms and potential harmful effects. To ensure their safe use in medical settings, it is vital to carefully determine the appropriate dosage, use controlled release methods, and select the optimal formulation [295,296]. AgNPs offer significant potential in wound healing applications due to their antibacterial, anti-inflammatory, and tissue-regenerative properties. Further investigation is necessary to improve their formulation, dosage, and delivery systems to ensure their safe and effective use in clinical settings.

### 4.2. AgNPs’ Antibacterial Properties

Extensive research has explored the antibacterial properties of AgNPs against various diseases. Numerous studies have investigated the production, description, and antibacterial effects of AgNPs obtained from different sources, including plant extracts and chemical techniques [204,235,297,298,299]. Various techniques, such as the disc-diffusion assay, determining the minimum inhibitory concentration (MIC), and evaluating the zone of inhibition, have been used to assess the antimicrobial efficacy of AgNPs. These studies have demonstrated that AgNPs effectively prevent the growth of Gram-positive and Gram-negative bacteria, including drug-resistant strains [235,236,237]. The antibacterial effect of AgNPs is multifaceted and complex. They can interact with bacterial cell membranes, leading to their breakdown and the release of cellular contents [238]. AgNPs can penetrate bacterial cells and interact with internal components, such as DNA, proteins, and enzymes, disrupting their structure and function [300]. Furthermore, AgNPs can generate reactive oxygen species (ROS), which can cause oxidative stress and damage bacterial cells [300]. The antibacterial activity of AgNPs has been observed to be influenced by their size, shape, and concentration. Smaller nanoparticles often have greater antibacterial effectiveness as a result of their bigger surface area and enhanced contact with bacterial cells [298].

It is crucial to recognize that while AgNPs have shown promise in combating bacteria, it is imperative to evaluate their potential impact on cells and the environment thoroughly. Extensive research has explored the compatibility of AgNPs with living organisms and their potential risks. It highlights the importance of precise dosing, regulated release, and appropriate formulation to ensure safe application [239].

### 4.3. Anti-Bacterial Mechanisms of AgNPs

1. Release of Silver Ions

Numerous studies have explored the antibacterial properties of AgNPs against a broad spectrum of harmful bacteria [235,297,298,299]. These nanoparticles are highly effective in killing bacteria because they generate silver ions, which can disrupt the bacterial cell membrane, impede enzyme activity, and hinder DNA replication [235]. The mechanism of action involves contact between the AgNPs and the bacterial cell wall, resulting in structural damage and cell death [238]. Multiple studies have demonstrated that AgNPs can effectively eliminate Gram-positive and Gram-negative bacteria, including drug-resistant ones. These nanoparticles have also demonstrated efficacy against various pathogens, such as *E. coli*, *Pseudomonas aeruginosa*, *Staphylococcus aureus*, and *Enterococcus faecalis* [235,236,298,301].

2. Interaction with the Bacterial Cell Wall:

Research has shown that AgNPs can harm the structure of bacterial cell walls and make the membrane more permeable, potentially leading to cell death [28,302] This occurs through the strong interaction of silver with thiol groups in respiratory enzymes within bacterial cells [303] Furthermore, AgNPs can cause the cell wall to become thinner and more permeable, destabilizing the peptidoglycan layer and releasing intracellular material and lysis of bacterial cells [302].

3. DNA Interaction

Studies have found that AgNPs can interact with DNA and affect cells and organisms differently. Specifically, AgNPs have been shown to cause DNA damage, such as strand breaks and chromosomal abnormalities [304,305], which can result in genotoxic effects such as broken DNA strands and chromosome defects [306,307]. Additionally, research by Maki et al. has demonstrated that AgNPs can cause DNA hypomethylation, leading to changes in gene expression [308]. Scientists have also explored the potential antiviral properties of AgNPs, including their effectiveness against herpes simplex virus and hepatitis B virus. The exact mechanisms by which AgNPs target viruses are still being studied, but researchers believe that interactions with viral DNA or RNA may hinder viral replication and infectivity [4].

4. Generation of Reactive Oxygen Species (ROS)

Several studies have shown that AgNPs can trigger the production of ROS in various biological systems [41,309,310]. ROS, including (O_2_^•^), hydrogen peroxide (H_2_O_2_), and hydroxyl radical (^•^OH), are highly reactive molecules that can cause oxidative stress and damage biological components such as DNA, proteins, and lipids [311]. Furthermore, researchers have explored the antibacterial properties of AgNPs and proposed that the generation of ROS plays a role in their ability to eliminate microorganisms [312]. By inducing oxidative stress in bacteria, the production of ROS by AgNPs can impair cellular constituents and ultimately lead to cell death [312].

Various methods can result in the generation of ROS by AgNPs. One involves the direct contact between these nanoparticles and biological components, producing ROS as a secondary product [311]. Another method involves the release of silver ions from the nanoparticles, which can stimulate ROS production through redox processes [41]. It has also been observed that the size of AgNPs can impact their ability to combat bacterial growth by influencing ROS production. Smaller AgNPs, in particular, have displayed a greater capacity to generate elevated levels of ROS compared to their larger counterparts, which may explain their increased antibacterial effectiveness [313].

5. Protein binding

The primary role of AgNPs is to attach to and penetrate bacterial cell membranes, which harms the membrane proteins. These proteins are essential for maintaining the structural integrity and permeability of the cell membrane, and disrupting them leads to cellular permeability and, eventually, the destruction of the bacterial cell [244,314].

Once inside the bacterial cell, AgNPs interact with various intracellular proteins, including enzymes. Many of these enzymes rely on thiol (–SH) groups for their activity, which AgNPs are strongly attracted to, inhibiting enzyme function [315]. This binding process interferes with crucial metabolic pathways in the bacteria, hindering their ability to survive and reproduce [314]. The primary function of AgNPs is to adhere to and infiltrate bacterial cell membranes. This interaction hurts the membrane proteins, which play a vital role in preserving the structural integrity and permeability of the cell membrane. The disruption of proteins causes cellular permeability and ultimately leads to bacterial cell demise [235,316].

### 4.4. Synergistic Effects with Antibiotics

**Figure 5:**
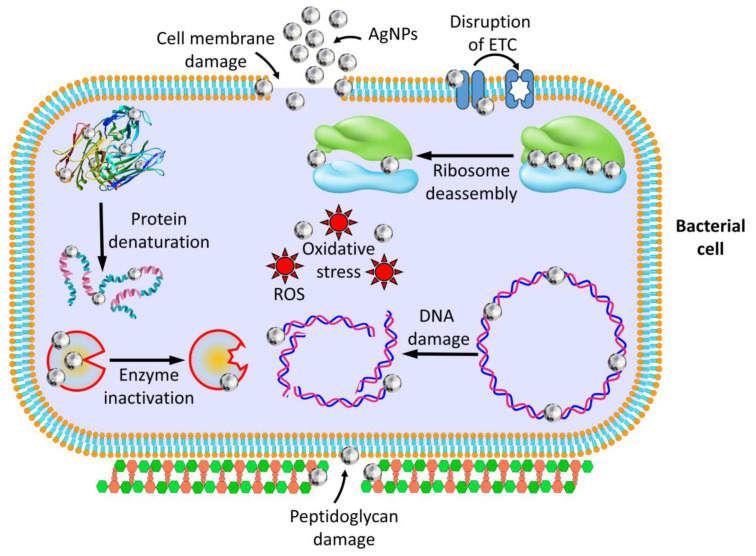
Antibacterial activity of silver NPs through different mechanisms.

Studies have been conducted to investigate the potential combined effects of AgNPs and antibiotics against various microorganisms, including bacteria (Figure 5). Results have shown that combining AgNPs with antibiotics has demonstrated greater antibacterial efficacy than using antibiotics alone [198,199]. This enhanced effect can be attributed to various factors:

Studies have been conducted to investigate the potential combined effects of AgNPs and antibiotics against various microorganisms, including bacteria (Figure 5). Results have shown that combining AgNPs with antibiotics has demonstrated greater antibacterial efficacy than using antibiotics alone [317,318]. This enhanced effect can be attributed to various factors:

1. Increased permeation: AgNPs can increase the permeation of antibiotics into microbial cells, allowing for higher concentrations of drugs within the cells and resulting in better effectiveness [318];
2. Inhibition of microbial resistance mechanisms: AgNPs can inhibit microbial resistance mechanisms, such as efflux pumps and biofilm formation, thereby increasing the susceptibility of microbes to antibiotics [318,319];
3. Increased ROS: AgNPs can amplify the creation of ROS within microbial cells, which induce oxidative stress and damage biological components, making bacteria more vulnerable to the effects of antibiotics [319];
4. Utilizing multiple cellular pathways: Combining AgNPs and antibiotics can effectively target multiple cellular pathways in microbes, resulting in a more comprehensive and potent antimicrobial impact [318].

It should be emphasized that the combined effects of AgNPs and antibiotics can differ based on the particular microbe, drug, and experimental circumstances. Additional investigation is required to enhance the combination methods and have a comprehensive understanding of the underlying mechanisms of synergy.

### 4.5. Anti-Biofilm Activity of AgNPs

AgNPs demonstrate several processes that inhibit biofilm formation. Their action disrupts the extracellular polymeric substance (EPS) matrix, compromising the biofilm’s structural integrity and enhancing bacterial cells’ vulnerability to antimicrobial drugs. AgNPs interfere with bacterial attachment to surfaces and impair quorum sensing, the cell signaling pathways that control biofilm development, preventing biofilm formation [320,321].

AgNPs can directly break the membranes of bacterial cells in biofilms, therefore causing heightened permeability and subsequent cell death. In addition, AgNPs produce ROS, which induce oxidative stress and harm to bacterial DNA, proteins, and lipids, further diminishing biofilms’ survival [322]. Algae AgNPs can improve the effectiveness of antibiotics by disturbing the biofilm matrix and enhancing drug penetration, hence successfully overcoming antibiotic resistance linked to biofilms [323].

Numerous studies have shown AgNPs’ potent antibiofilm properties against various bacteria. Lara et al. conducted a study to investigate the effects of AgNPs on *Candida albicans*’ biofilms. The study found that AgNPs could prevent the growth of *C. albicans* biofilms and break down already-established biofilms [245]. Similarly, Yin et al. examined AgNPs’ use to prevent dental caries and found that smaller nanoparticles were more effective in infiltrating biofilms than larger ones [244]. Mohanta et al. focused on AgNPs’ antibacterial and antibiofilm properties against *S. aureus* and *E. coli*. The study found promising results for the efficacy of AgNPs against these harmful microorganisms [200].

Recent studies have shed light on the potential of AgNPs to regulate the growth of antibiotic-resistant bacteria that form biofilms. One study found that AgNPs could suppress biofilm production in these bacteria [246] In addition, research has shown that AgNPs have antimicrobial properties and can effectively eliminate streptococcal biofilms, making them a promising antibiofilm agent in oral healthcare [247]. The unique characteristics of AgNPs, including their size, ability to penetrate biofilms, and impact on antibiotic-resistant bacteria, make them a valuable tool in the fight against biofilm-associated infections.

### 4.6. Antiviral Activity of AgNPs

Studies have shown that AgNPs have antiviral properties against a range of viruses, including coronaviruses, respiratory syncytial viruses, influenza viruses, herpes simplex virus, and hepatitis B virus [4,215,241,253,324]. AgNPs use various mechanisms to fight viruses, such as preventing viruses from entering host cells and disrupting viral replication [215,242]. While the relationship between AgNPs and viruses is still being investigated, it is clear that these nanoparticles have a unique antiviral mechanism that may differ depending on the virus being targeted. For example, research has shown that AgNPs can combat HIV-1 by acting as virucidal agents or inhibitors of viral entry during the initial stages of viral replication [242].

The antiviral properties of AgNPs are attributed to their ability to cause oxidative stress and produce ROS, which can destroy viral structures and prevent viral attachment and entry into host cells [215,242].

In addition, studies have explored the potential synergistic effects of AgNPs with antiviral medications. For example, the combination of AgNPs and oseltamivir has been shown to effectively inhibit the H1N1 influenza virus [215], while curcumin-modified AgNPs have demonstrated strong suppression of respiratory syncytial virus infection [241].

### 4.7. Anti-Fungi Activity of AgNPs

AgNPs have exhibited substantial fungicidal effects against several plant pathogenic fungi, clinical pathogens, and phytopathogenic fungi. In a study conducted by Kim et al., the antifungal effectiveness of AgNPs against plant pathogenic fungi was evaluated. The researchers discovered that the nanoparticles hindered the growth of the fungi, suggesting their potential as agents for combating fungal infections [325].

A study investigated the antifungal properties of AgNPs against drug-resistant opportunistic fungus commonly found in immunocompromised persons. The study confirmed the efficacy of AgNPs in treating drug-resistant fungal infections [326]. Shi et al. investigated the antibacterial properties of biocompatible silver microspheres and their possible application in the treatment of fungal keratitis. Their focus was on the utilization of various AgNPs for treating skin diseases and phytopathogenic fungi [327]. A study assessed the impact of chitosan-silver-copper nanocomposites on *C. albicans*. They also investigated the lowest concentrations at which these nanocomposites inhibited the growth of *C. albicans* and the lowest concentrations at which they killed the fungus [328]. In addition, studies have presented compelling evidence supporting the effectiveness of AgNPs in combating a broad range of fungi, including dermatophytes and non-dermatophytic fungi [329,330].

### 4.8. Anti-Parasite Activity of AgNPs

Studies have shown that AgNPs hold promise in combating parasites. Marimuthu et al. conducted a study using green methods to manufacture AgNPs using the aqueous leaf extract of Mimosa pudica. The study targeted the larvae of the malaria vector Anopheles subpictus, the filariasis vector Culex quinquefasciatus, and Rhipicephalus microplus and demonstrated their efficacy [254]. Palanisamy et al. (2024) also displayed the potential of AgNPs as potent anti-parasitic drugs, showcasing their larvicidal efficacy against Passiflora foetida [331]. Recent studies by Souza et al. (2022) and Zein et al. (2022) uncovered AgNPs’ anti-leishmanial and anti-trypanosome properties [255,332]. Furthermore, Gajera et al. (2023) and Goel et al. (2020) demonstrated the broad effectiveness of these nanoparticles against parasites, showcasing their anthelmintic properties against Caenorhabditis elegans and gastrointestinal helminths, respectively [333,334].

### 4.9. Immune System

AgNPs have attracted attention for their ability to trigger immunological reactions within the body’s immune system. Studies have shown that AgNPs can activate important components such as macrophages, dendritic cells, and lymphocytes, which play a crucial role in defending against infections [7].

Interestingly, exposure to AgNPs has been associated with the production of cytokines, which are essential signaling molecules that regulate immunity and inflammation. Furthermore, recent research by Shalaby et al. (2022) suggests that AgNPs can enhance wound healing by reducing cytokine secretion, mast cell infiltration, and lymphocyte presence [335]. Moreover, AgNPs have been found to interact with macrophages and stimulate the production of cytokines such as interleukin-8 (IL-8) [336]. While AgNPs have demonstrated their ability to modulate the immune system, it is essential to note that excessive cytokine generation can lead to a cytokine storm, which has negative consequences [337].

In laboratory settings, AgNPs have been found to cause changes in the immune system, resulting in antiviral and immune-modulating effects when tested using respiratory syncytial virus (RSV) infection as a model [338]. Furthermore, other studies have shown that AgNPs possess hepatoprotective properties, which help maintain cytokine levels and reduce inflammatory pathways [339].

Extensive research has been conducted on the behavior of AgNPs and their impact on the natural immune system, specifically the interferon-induced guanylate-binding proteins (GBPs) [340]. GBPs are crucial for the innate immune response against viral infections and are activated by interferons and pro-inflammatory cytokines. Their primary function is to regulate intracellular infections [340]. While the interaction between AgNPs and GBPs has yet to be thoroughly explored, AgNPs can influence the activity of GBPs or affect their synthesis in response to viral infections. Further investigation is necessary to fully understand the interaction mechanisms between AgNPs and GBPs and their potential impact on antiviral immune responses.

### 4.10. Drug Delivery

The distinctive characteristics and prospective uses of AgNPs have led to their increased recognition in the drug delivery domain. Recent studies have shown that AgNPs can effectively deliver medication by modifying their surface with ligands, antibodies, or peptides that attach selectively to target cells [341,342]. This improves the efficacy of the medicine while minimizing any unwanted side effects. Nanoparticles provide multiple benefits such as their compact dimensions, extensive surface area, and adjustable surface chemistry, rendering them appropriate for enclosing and transporting diverse medicinal substances [5].

An important benefit of AgNPs in drug delivery is their capacity to improve the stability, solubility, and bioavailability of medicines [5]. They have the ability to act as carriers for both hydrophobic and hydrophilic medicines, safeguarding them from degradation and enhancing their transportation to specific locations. Moreover, AgNPs have the capability to be modified with ligands or targeting moieties in order to precisely transport medications to specific tissues or cells [5].

Scientists have devised a novel technique for precise targeting using peptide-modified AgNPs. These specially modified AgNPs are incorporated into producing short peptide amphiphile (sPA) nanosystems. The sPA nanosystems possess a superior capacity to interact with bacterial cell surfaces, facilitating better distribution throughout the body and improved absorption into tissues. Consequently, the antibacterial efficacy of these AgNPs is significantly increased [343].

The possibility of AgNPs in controlled drug release systems has also been investigated. By altering the surface characteristics of the nanoparticles, it is possible to manage the release of medications, enabling a prolonged and regulated delivery of medication [5]. This can enhance the therapeutic effectiveness of medications and minimize adverse effects.

In addition, AgNPs have demonstrated the potential to surmount biological obstacles, such as the blood-brain barrier, in order to transport medications to the central nervous system [273]. They may be manipulated to traverse these obstacles and transport therapeutic substances to precise locations, so creating novel opportunities for the treatment of neurological illnesses. AgNPs have natural antibacterial qualities in addition to their ability to carry drugs. This can be beneficial in fighting infections at the location where drugs are administered [341]. They possess the ability to inhibit bacterial proliferation and mitigate the likelihood of infection linked to implanted devices or surgical interventions.

### 4.11. Cancer Treatment

AgNPs demonstrate anticancer properties in multiple ways. They can trigger apoptosis in cancer cells by stimulating both intrinsic and extrinsic mechanisms, resulting in mitochondrial malfunction and the activation of caspases. AgNPs also regulate the transcription of pro-apoptotic and anti-apoptotic proteins, facilitating predetermined cell death in cancer cells [344,345].

Moreover, AgNPs have the potential to disrupt crucial signaling pathways that control the growth and survival of cancer cells, including Akt, ERK, and NF-κB cascades. This disruption diminishes the rate of cell division and hampers tumor development. AgNPs can induce DNA damage, resulting in cell cycle arrest and an induction of apoptosis or necrosis in cancer cells [293]. This potential to disrupt cancer cell survival mechanisms is a fascinating aspect of AgNPs in cancer treatment.

AgNPs have anti-angiogenic effects by suppressing the development of essential blood vessels required for tumor proliferation. By inhibiting pro-angiogenic signals such as VEGF, AgNPs can decrease the blood flow to tumors, restricting their proliferation and ability to spread to other body parts. AgNPs exhibit coupled processes that render them a desirable contender for anticancer treatments [346].

Extensive research has explored the potential of using AgNPs in cancer research and treatment. While some studies have shown promising results, it is essential to note that this field is still evolving and requires further investigation to fully understand the potential benefits and risks of employing AgNPs in cancer-related applications.

One promising study area involves using AgNPs for cancer diagnosis and imaging. Alavijeh et al. demonstrated that AgNPs can be customized with targeting ligands or imaging agents to locate cancer cells or tumors selectively. This targeted approach enhances the detection and imaging of malignant tissues [263]. By utilizing this strategy, the accuracy of cancer detection can be improved, and the effectiveness of treatment can be tracked.

Moreover, AgNPs have also been explored for their potential use in cancer treatment and diagnostic purposes. They have been investigated as anti-cancer drugs, showing promising results in suppressing the proliferation of colon cancer cells [264]. Research has indicated that AgNPs can exhibit cytotoxic properties on cancer cells, leading to either cell death or suppression of tumor growth [305]. The underlying mechanisms of action are believed to involve the production of ROS, interference with cellular activities, and initiation of apoptosis [305].

### 4.12. AgNPs Coating Surfaces

Extensive research has been conducted on the potential uses of AgNPs for coating various surfaces. These coatings have been shown to possess numerous benefits, including antibacterial properties, enhanced thermal features, improved catalytic activity, and surface modifications. A recent study explored the production of composites composed of silver bromide nanoparticles and polymers, revealing their potent antibacterial effects against bacteria found in water and air [347]. Additionally, investigations of AgNP coatings on biopolymers have primarily focused on their antibacterial and antiviral properties. A recent study indicated that applying AgNPs on biopolymer pellets leads to the creation of a surface with both antibacterial and antiviral characteristics [279].

Recent studies in materials science have explored ways to enhance the antibacterial properties of porous titanium surfaces. One promising technique involves applying a layer of AgNPs using electron-beam evaporation. Researchers have found this approach is simple and effective, as demonstrated in a recent study [280]. Additionally, scientists have investigated superhydrophobic coatings and their potential applications. For instance, a study examined the impact of various solvents on polyurethane coatings that contain hydrophilic SiO_2_ nanoparticles. The researchers showed how inorganic nanoparticles, including AgNPs, can be used to create uneven surfaces on superhydrophobic coatings, as reported in a separate study [281].

A promising new method for creating an antibacterial coating that does not rely on the surface has been proposed. This innovative approach combines a sticky protein from mussels with a peptide that binds to silver. Doing so makes it possible to create AgNPs with a broad spectrum of antibacterial properties. As a result, this technique is highly versatile and can coat various surfaces [282]. Researchers have also looked into applying AgNPs to zeolite, sand, fiberglass, and resin substrates to eliminate pathogenic bacteria in groundwater. Their findings revealed that filter materials coated with AgNPs effectively eradicate harmful microorganisms present in groundwater [283].

### 4.13. Food Industry

The antibacterial potential of AgNPs has piqued the interest of the food industry. These nanoparticles have demonstrated their ability to inhibit many bacteria that cause illness and spoil food [267]. To enhance the microorganism-killing capabilities of materials used in food packaging, coatings and films containing AgNPs have been developed [268,269] For instance, AgNP-infused polyethylene films have been created for food packaging purposes, which have been shown to slow down microorganism growth [348].

Moreover, researchers have explored the potential of AgNPs in water disinfection for food processing and preparation. Given their potent antibacterial properties, these nanoparticles effectively eliminate microorganisms and ensure water safety [270,271]. Regulatory agencies have evaluated the safety of AgNPs in food contact products. According to the European Food Safety Authority (EFSA), using AgNPs in polymers is safe as long as they are utilized within specific migration limits [270].

### 4.14. AgNPs Coating Implants

AgNPs have shown great potential in implants due to their unique attributes, including biocompatibility, improved surface features, and antibacterial capabilities. Numerous investigations have been conducted on implants coated with AgNPs, including dental, bone, and titanium implants.

A recent study examined the antibacterial mechanism of AgNPs and their potential use in dentistry. The study found that when AgNPs are integrated into a material, they consistently come into direct contact with bacterial cells, inhibiting their growth and exhibiting potent antibacterial properties [244]. Another investigation showed that AgNP-infused hydroxyapatite (HAp) scaffolds displayed distinctive antibacterial properties, effectively inhibiting bacterial infections associated with bone implants [2]. Research has demonstrated that implants coated with AgNPs can enhance antibacterial effectiveness while causing minimal harm to regular human cells [274]. Also, the targeted use of AgNPs on dental implants restricts their antibacterial effects to the immediate vicinity of the implant. This focused intervention safeguards the adjacent indigenous microbiome by restricting the contact of commensal microorganisms with the silver nanoparticles, therefore mitigating the potential for disturbing the general microbial equilibrium in the mouth [349].

A thorough examination has been conducted on the effects of titanium implants coated with AgNPs, focusing on their antimicrobial and toxic properties. The research shows that AgNPs on dental implant surfaces decrease harmful effects on cells while providing long-lasting germ-fighting capabilities [275]. Additionally, introducing silver ions onto TiNb-based alloy surfaces has shown remarkable antibacterial efficacy [276]. Plasma immersion ion implantation is another method employed to fixate AgNPs onto titanium surfaces, resulting in a durable antibiofilm effect against bacteria sticking to the surface. This method prevents the development of drug-resistant bacteria and cytotoxicity [350].

### 4.15. AgNPs Activity Biofouling

AgNPs have shown great potential in effectively combating biofouling in various applications. Their antibacterial properties effectively stop the growth and adhesion of microbes [39]. To further enhance their antifouling properties, AgNPs have been integrated into hydrogels and coatings [351], which have shown the ability to inhibit bacterial adhesion, biofilm development, and fouling [352]. Additionally, incorporating AgNPs into nanocomposite membranes has improved their antifouling properties [352]. Using AgNPs in these applications is a practical approach to counteracting biofouling and hindering the adherence and proliferation of microorganisms. However, concerns about their toxicity to aquatic life and the potential accumulation of silver ions in the environment are valid. Furthermore, the long-term effects of continuous low-level silver exposure on the environment and human health still need to be fully understood.

## 5. Host Cell Defense Strategies against AgNP-Induced ROS and Clustering

In order to shield host cells from the clustering and formation of ROS by AgNPs, a combination of targeted delivery, cellular defenses, and regulated nanoparticle characteristics is employed.

Surface modification of AgNPs with ligands or antibodies that selectively bind to receptors overexpressed on cancer cells or pathogens enables their selective absorption. Adopting this focused strategy minimizes the interaction between AgNPs and healthy cells, decreasing the likelihood of accidental clustering and formation of ROS in non-target tissues [353,354].

The host cells have comprehensive antioxidant defense mechanisms, which include enzymes such as superoxide dismutase (SOD), catalase, and glutathione peroxidase. These enzymes effectively counteract ROS and prevent oxidative damage. Antioxidants enhance cellular homeostasis by quickly neutralizing ROS produced around or inside healthy cells, reducing oxidative stress and preserving cellular integrity [355].

Furthermore, the possible harmful effects of AgNPs are controlled by making their concentration, size, and duration of exposure optimal in therapeutic environments. Lower concentrations of AgNPs decrease the probability of excessive generation of ROS and the aggregation of nanoparticles surrounding healthy cells [356,357]. Furthermore, using surface coatings containing biocompatible materials, such as polyethylene glycol (PEG) or other polymers, might decrease the aggregation and reactivity of nanoparticles, reducing their harmful impact on host tissues [358].

## 6. AgNPs Toxicity Issues

It is crucial to recognize that while AgNPs possess antibacterial and antiviral properties, it is essential to thoroughly assess their potential negative impacts on both human health and the environment. The environmental impact of AgNPs has been a significant area of research, with studies highlighting the possible consequences of their alteration and adverse effects on different ecosystems [244,359,360]. Concerns about their potentially harmful effects and tendency to accumulate in aquatic creatures underscore the need to comprehensively evaluate their environmental impact [361,362,363]. Bioaccumulation of AgNPs in various fish organs has been documented by researchers, suggesting potential detrimental impacts on aquatic organisms [364]. Additionally, scientific research has shown that AgNPs may harm animal reproductive systems, including negative implications on reproductive performance, semen quality, and sex hormones [365].

The effects of AgNPs on human health are a complex and multifaceted issue. Studies have linked their presence to various adverse effects, including inflammation, oxidative stress, and toxicity in cultured cells [366]. Extensive research has been conducted to evaluate their impact on human organs, and the results have revealed the potential harm they can cause to various organs and species, such as cell death, genetic material damage, and adverse effects on well-being. The toxicity of AgNPs has been attributed to the release of free silver ions, which can cause cellular toxicity and harm several organs [244,366].

Additionally, studies have shown that AgNPs may have adverse effects on human health, such as liver and kidney toxicity, and possible impacts on reproduction and respiration [367]. Due to their potentially harmful effects on human health, concerns have been raised about using AgNPs in consumer goods and medical therapies [368]. Moreover, research has demonstrated that AgNPs can induce toxicity in the reproductive, pulmonary, and cutaneous systems [369].

The use of AgNPs has been found to trigger oxidative stress in cells, potentially leading to the development of diseases, including neurological diseases and genetic mutation. Recent studies have demonstrated that exposure to AgNPs can cause cellular damage and increase the risk of neurological illnesses [158]. Furthermore, the impact of AgNPs on human cells has been assessed, uncovering potential harm to the genetic material and the presence of oxidative stress at specific levels of concentration [158]. At certain concentration levels, AgNPs have also been linked to harm to genetic material and oxidative stress [158,370], and genotoxic and cytotoxic effects have been observed [370,371]. To properly evaluate the toxicity of AgNPs in humans, a multidisciplinary approach is necessary, considering factors such as exposure levels, potential health risks, and long-term consequences [372].

## 7. Barriers to Clinical Use

Despite the promising characteristics of AgNPs, their transition from experimental study to widespread use in clinical settings is impeded by a variety of obstacles and limitations. This underscores the crucial need for further research and standardization in this field.

An important issue related to the therapeutic application of AgNPs is the stability and consistency of AgNP production. The physiological activity of AgNPs is greatly influenced by their physical and chemical characteristics established by the production process. Diverse chemical, biological, or physical approaches can generate nanoparticles with varying dimensions, geometries, and surface properties, leading to uneven biological impacts [373]. Furthermore, AgNPs are susceptible to aggregation, which can modify their size distribution and surface characteristics, diminishing their efficiency level. Ensuring the stability of AgNPs and limiting their aggregation is essential for preserving their intended functioning in therapeutic applications [374]. Therefore, achieving consistency in synthesis is a significant obstacle that must be surmounted to guarantee predictable and dependable therapeutic results.

Regulations governing AgNPs also provide significant obstacles. Securing regulatory clearance for novel clinical applications necessitates thorough evidence of the safety, effectiveness, and strict quality control measures. Nevertheless, the absence of standardized procedures for assessing these factors adds complexity and prolongs the regulatory procedure. Distinct regulatory authorities may have divergent criteria, adding complexity to the approval procedure [375,376].

The interaction of AgNPs with biological systems introduces further complexity. AgNPs have the ability to interact with a diverse range of biomolecules, such as proteins, lipids, and DNA, leading to the formation of a structured protein corona [377]. The presence of this protein corona can significantly alter the behavior of the nanoparticle, affecting its stability, biodistribution, and cellular absorption. The unpredictable nature of the interactions involved in creating AgNPs for specific therapeutic applications presents a challenge, as they can lead to unforeseen biological consequences [377,378]. Moreover, there is concern about the potential immunogenicity of AgNPs. Their mere presence could trigger an immunological response, leading to inflammation or other adverse effects mediated by the immune system [379,380].

## 8. AgNPs’ Specificity and Selectivity

AgNPs are adaptable substances that exhibit diverse biological activities, such as antibacterial solid effects against different pathogens, anti-cancer characteristics, and uses in wound healing. Nevertheless, a notable drawback of AgNPs is their lack of selectivity, which might result in non-selective cytotoxicity and possible adverse consequences in healthy tissues [142,376]. Improving the targeting precision of AgNPs is a crucial focus for enhancing their specificity for different therapeutic uses. Various approaches are being investigated to achieve this.

An auspicious approach to enhance the selectivity of AgNPs is surface functionalization. This process entails altering the surface of nanoparticles by adding ligands, such as antibodies, peptides, or small molecules, which can identify and attach to specific receptors on target cells [353,381]. For instance, by conjugating AgNPs with folic acid, which specifically targets the folate receptors strongly expressed in numerous cancer cells, the selectivity of these nanoparticles for malignant tissues can be improved, while normal cells remain unaffected [382,383]. Such precise delivery methods can minimize unintended consequences and enhance the therapeutic effectiveness of treatments based on silver nanoparticles.

Enhancing the specificity of AgNPs presents a significant potential for targeted medication delivery. By conjugating therapeutic substances, such as chemotherapeutic drugs or antibiotics, to nanoparticles and then coating them with targeting molecules, it becomes feasible to precisely guide AgNPs towards impaired cells or tissues. This method reduces the impact on normal cells and systemic adverse effects, making it particularly attractive for use in oncology and infectious diseases [384,385]. Moreover, the incorporation of controlled release systems, where AgNPs are included in biodegradable matrices that release their contents in response to specific stimuli, provides additional levels of specificity. These stimuli-responsive systems can be tailored to release AgNPs in the acidic environment of tumors or inflamed tissues, ensuring targeted therapeutic effects at the intended site [386,387].

Developing responsive AgNPs is a novel method to improve specificity. Nanoparticles specifically engineered to release their therapeutic molecules when exposed to specific wavelengths of light enable accurate manipulation of their activity in space and time. This degree of control is very advantageous in applications requiring localized therapy, such as in certain malignancies or localized infections [138,388].

There is ongoing research into the use of AgNPs as carriers for gene editing and RNA interference treatments. By attaching gene-editing tools such as CRISPR-Cas systems or small interfering RNA (siRNA) to AgNPs, scientists can achieve precise and targeted genetic engineering. This approach holds significant promise for the treatment of genetic diseases and malignancies [389,390].

## 9. Challenges and Future Directions for AgNPs

One of the significant challenges surrounding AgNPs is the possibility of toxicity and safety concerns. While the properties of these nanoparticles show great potential, it is crucial to thoroughly evaluate their impact on human cells and the environment [391,392]. This includes examining any potential harm that they may cause.

Regulating and standardizing the use of AgNPs poses significant challenges due to the complex nature of evaluating associated risks. These risks vary in size, shape, surface characteristics, and quantity. The fast-paced advancements in nanotechnology further complicate regulatory efforts to keep up with the latest developments. The interdisciplinary nature of nanotechnology, which spans biology, chemistry, physics, and engineering, adds to the complexity of standardization and regulation. Moreover, the regulatory system must also consider ethical, social, and long-term implications when dealing with nanomaterials [393,394].

Environmental stability and transformation are crucial factors to consider when evaluating the potential risks associated with AgNPs. These nanoparticles are susceptible to environmental changes that can alter their properties, affecting their movement and potentially harming the environment [395]. Therefore, understanding the stability and transformation of AgNPs under different environmental conditions is essential for developing effective risk management strategies.

Antibiotic resistance is a growing concern, and while AgNPs have shown promise in combating resistant microbes, it is crucial to remain vigilant against the possibility of resistance to these nanoparticles. To ensure their continued effectiveness as antibacterial agents, more research is needed to better understand how AgNPs work and the potential resistance pathways [393].

The cost and scalability of producing AgNPs for various applications can pose challenges. It is imperative to devise synthesis techniques that are economically viable and can be scaled up to enable their widespread use in multiple industries, including wound healing [394].

## 10. Conclusions

The unique properties and potential applications of AgNPs have captured the attention of various fields. With their remarkable ability to combat bacteria, viruses, and fungi, these nanoparticles are highly valuable for wound healing. They prevent infections and accelerate tissue regeneration, making them indispensable for wound dressings, hydrogels, and other delivery methods. However, the benefits of AgNPs extend beyond wound healing. They can potentially revolutionize cancer therapy by facilitating precise medication delivery and imaging. In addition, they can serve as protective layers for medical devices and as agents for inhibiting the formation of biofilms in water treatment and dental materials.

Nevertheless, several obstacles must be addressed before AgNPs can be widely adopted, including concerns about cell toxicity, consistent standards, regulations, stability maintenance, antibiotic resistance, cost-effectiveness, scalability, combination therapies, and environmental impact. With further research and progress, AgNPs have the potential to offer significant advantages in wound healing and antimicrobial treatments. Collaborative efforts can enable us to effectively harness the opportunities’ full potential and tackle any associated obstacles.

Biologically produced AgNPs are commonly distinguished by their reduced dimensions and even dispersion, which can be ascribed to the utilization of natural reducing agents, such as enzymes and proteins. These nanoparticles are conjugated with biomolecules that augment their stability and compatibility with living organisms, rendering them appropriate for biomedical uses such as antibacterial, antifungal, antiviral, biosensing, drug delivery, and wound healing. Biochemical synthesis offers two main benefits: its environmentally benign character, as it does not include the use of toxic chemicals, and the improved biocompatibility of the nanoparticles produced. Nevertheless, this approach is frequently linked to intricacy in the synthesis procedure, obstacles in regulating the dimensions and morphology of the nanoparticles, and issues in expanding large-scale industrial manufacturing.

In contrast, chemical techniques such as chemical reduction and photochemical synthesis provide more precise manipulation of the size, shape, and uniformity of AgNPs, rendering them adaptable for many uses. The simplicity and scalability of these techniques make them advantageous for large-scale manufacturing in industrial settings. Nevertheless, using hazardous substances as lowering or stabilizing agents has notable disadvantages, such as possible toxicity and environmental complications, which restrict their application in biological domains. Furthermore, chemically produced AgNPs find widespread application in catalysis, electronics, textiles, and antimicrobial coatings because of their adjustable characteristics and convenient mass manufacturing.

## Figures and Tables

**Table 2:** Physical characterization techniques for AgNPs.

Technique	Property Characterized	Description	References
UV-Vis Spectroscopy	Optical Properties	Analyzes the absorption and scattering of light, which can be related to the size and concentration of the nanoparticles.	[1]
Transmission Electron Microscopy (TEM)	Size, Shape, Morphology	High-resolution images to determine the size and shape at the nanoscale.	[159]
Scanning Electron Microscopy (SEM)	Surface Morphology	Provides surface detail and composition information.	[160]
X-ray Diffraction (XRD)	Crystal Structure	Identifies the crystalline phases and orientation, providing insights into the structural properties.	[161]
Energy-Dispersive X-ray Spectroscopy (EDS)	Elemental Composition	Provides elemental analysis and chemical characterization.	[162,163]
Dynamic Light Scattering (DLS)	Hydrodynamic Size	Measures the size distribution of particles in suspension based on light scattering.	[164,165]
Atomic Force Microscopy (AFM)	Surface Topography	Gives a 3D profile of the surface at the nanoscale.	[166]
Zeta Potential Analysis	Surface Charge	Determines the surface charge and stability of nanoparticles in suspension.	[167]

**Table 3:** An in-depth analysis of AgNPs and their potential uses.

Application Area	Description	Clinical Insights	Setting	References
Antibacterial	AgNPs are used in coatings and textiles to inhibit bacterial infections, especially against antibiotic-resistant strains.	AgNPs, in combination with antibiotics, enhance effectiveness against multidrug-resistant bacterial infections.	In vitro	[235,236,237,238,239,240]
Antiviral	AgNPs demonstrate antiviral capabilities against viruses such as coronaviruses, HIV-1, influenza, herpes simplex, and hepatitis B.	AgNPs act as virucidal agents and show synergistic effects with antiviral drugs such as oseltamivir against H1N1.	In vitro	[215,241,242,243]
Antibiofilm	AgNPs are incorporated into coatings to prevent biofilm formation and disrupt established biofilms.	Effective against antibiotic-resistant bacteria biofilms, such as those formed by *Staphylococcus aureus* and *Escherichia coli*.	In vitro	[200,244,245,246,247,248]
Antifungal	AgNPs exhibit antifungal properties against various fungi, including those causing plant diseases and infections in immunocompromised individuals.	Used to treat drug-resistant fungal infections and enhance the effectiveness of antifungal treatments.	In vitro	[38,40,246,247,249,250,251,252,253]
Antiparasitic	AgNPs show potential against parasites such as malaria vectors, leishmaniasis, and Acanthamoeba.	Demonstrate efficacy against parasites at different lifecycle stages when produced using various green synthesis methods.	In vitro	[254,255,256,257,258,259,260,261,262]
Anticancer and Antitumor	AgNPs induce cytotoxicity in cancer cells and enhance targeted drug delivery.	Used alone or with other anticancer drugs, they increase the therapeutic efficacy of treatments such as chemotherapy.	In vitro	[5,263,264,265,266]
Food Industry	AgNPs are used in the food industry for their antibacterial properties, including food packaging, coatings, and films to prevent food spoilage and contamination.	AgNPs enhance the antimicrobial properties of food packaging materials, helping to prevent the growth of drug-resistant bacteria in food products.	In vitro	[267,268,269,270,271,272]
Drug Delivery	AgNPs improve drug stability and bioavailability, offering controlled release for enhanced treatment efficacy.	Can penetrate biological barriers, such as the blood-brain barrier, facilitating targeted drug delivery.	In vitro	[5,273]
Medical Implants	AgNPs are used to coat medical implants, enhancing their antibacterial properties and longevity.	Reduce infection rates in implanted devices, show minimal cytotoxicity, and promote osseointegration.	In vitro	[274,275,276,277,278]
Surface Coatings	AgNPs in surface coatings offer antibacterial properties and thermal enhancements, effectively fighting bacteria in air and water…	AgNPs enhance the antibacterial properties of surfaces, including porous titanium, and can modify surfaces for better microbial resistance.	In vitro	[99,279,280,281,282,283,284,285]
Dentistry and dental industry	AgNPs are utilized in dental materials to prevent biofilm formation, crucial for oral health.	Improve antibacterial properties in various dental applications, supporting sterile environments.	In vitro and In vivo	[244,286,287,288,289]
